# Guided Bone Regeneration with Occlusive Titanium Barrier: A Case Report and Clinical Considerations

**DOI:** 10.3390/biomimetics8010106

**Published:** 2023-03-06

**Authors:** Lucio Milillo, Massimo Petruzzi

**Affiliations:** 1 Private Practice in Bari, Via Jatta 15, 70100 Bari, Italy; 2 Interdisciplinary Department of Medicine, University of Bari “Aldo Moro”, 70124 Bari, Italy; 3 Department of Restorative, Preventive and Pediatric Dentistry, University of Bern, 3012 Bern, Switzerland

**Keywords:** blood clot, titanium foil, bone augmentation, guided bone regeneration, dental implants, socket preservation

## Abstract

The need to obtain adequate bone volumes for prosthetic rehabilitation supported by implants, using different techniques and materials, represents an urgent need in modern dentistry. We report a case regarding the management of implant-prosthetic rehabilitation of the first and second upper right molars, in which no less than 4 mm of crestal bone remained to insert two implants. Regeneration of the residual bone was previously performed using a customized titanium barrier and a filler of a blood clot with tricalcium beta phosphate. The bone gain (3 mm) was evaluated by comparing CBCT images, while the implant stability (mean 70) was assessed with the ISQ measurement. A regenerated bone sample was taken for histological analysis. Guided bone regeneration obtained with a titanium barrier and blood clot allowed for the insertion of stable implants in a mature bone without heterologous material.

## 1. Introduction

Rehabilitation of post-extractive alveolar sites with dental implants requires a careful approach in the diagnosis and surgical phases. In selected cases, guided bone regeneration (GBR) is necessary to achieve adequate bone volume and complete implant osteointegration [1].

New GBR surgical techniques have been proposed in the last five years in accordance with the development of new biomaterials [2,3,4,5,6,7,8,9,10].

Among these new techniques, using occlusive barriers in titanium has been proposed to offer a rigid protective shell containing the graft material and avoid the collapse and invasion of soft tissues in the regenerated site. The barriers also guarantee protection from bacterial contamination [11,12,13].

Among the existing fillers used under the occlusive barriers [14,15], blood clots mixed with beta-tricalcium phosphate seems to be the most suitable [16].

We present a case of GBR in the maxillary bone molar zone using a customized occlusive titanium barrier and beta-tricalcium phosphate as filler material. The aim was to demonstrate the effectiveness of this technique for increasing bone volume, bone quality, and implant stability.

## 2. Case Report

**Figure 1:**
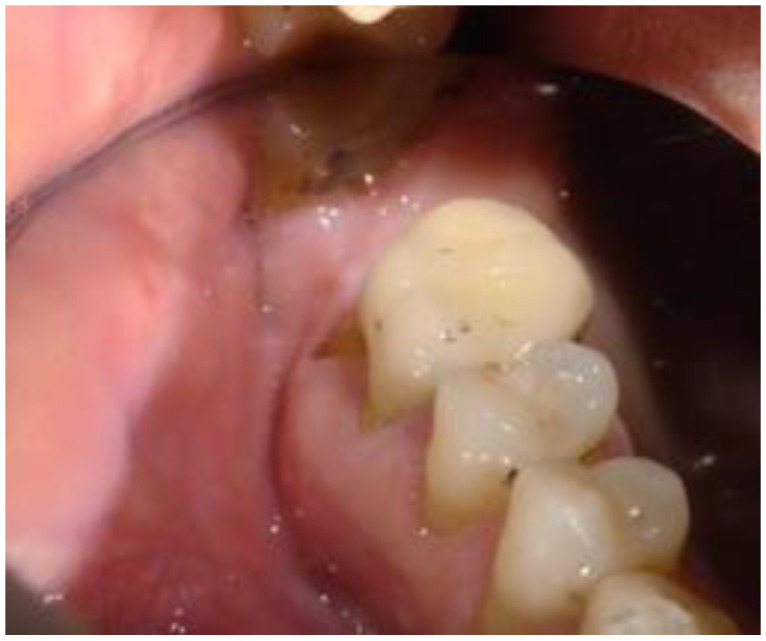
The preoperative site shows the absence of 1.7 and the crown on 1.6 (with abscess).

A 48-year-old Caucasian male was presented to our attention for a recurrent abscess on tooth 1.6. The patient was in good health, was a non-smoker, and had good control of oral hygiene. The intraoral examination showed an optimal state of oral health. There were three missing teeth (1.7, 2.8, and 3.6; Figure 1) and a metal-ceramic crown on 1.6. The periodontal probing on 1.6 evidenced a probing depth of 12 mm in correspondence with the mesial and palatal root, from mesial to distal.

The panoramic X-ray examination showed a previous endodontic treatment and a radiolucency on 1.6 that affected the apex of the buccal root and furcation.

Analyzing the cone beam computed tomography (CBCT) and the 3D reconstruction superimposing a diagnostic wax-up of the bone model obtained with Digital Imaging and Communications in Medicine (DICOM) files, it was decided to preliminarily use GBR for the insertion of the implants. The goal of GBR was to obtain a ridge augmentation, which concerned teeth 1.7 and 1.6.

### 2.1. Clinical and Surgical Procedures

The patient was prescribed an antibiotic therapy of amoxicillin (875 mg) with clavulanic acid (125 mg) that started two days before surgery. The surgery was performed under local anesthesia for infiltration with 1:100 articaine.

Two 5 mL tubes of peripheral blood were collected, which coagulated without any manipulation (centrifugation and/or addition of other drugs), and which provided the clot/scaffold to be inserted into the titanium barrier together with the beta-tricalcium phosphate. The proportion adopted was 1 gr. of beta-tricalcium phosphate per 5 mL of clot.

Tooth 1.6 was extracted, showing the presence of a fiberglass pin that protruded from the apex of the palatal root.

**Figure 2:**
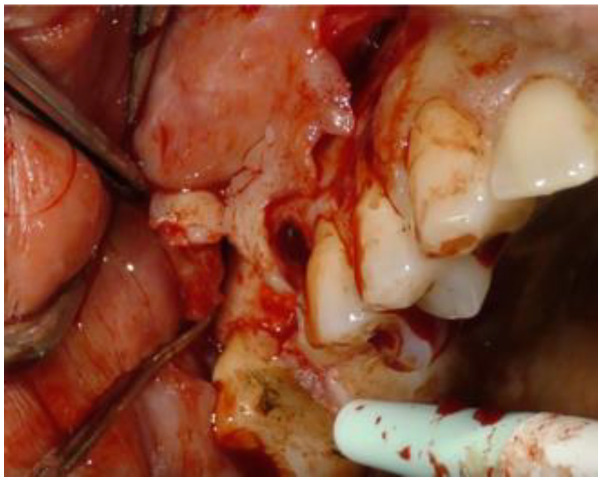
Detail of the design of the vestibular flap.

The surgery required the design of a full-thickness vestibular trapezoidal flap, extending from the mesial papilla of tooth 1.8 to the mesial papilla of tooth 1.4. The incision was performed with a discharge in the direction of the fornix in a distal direction starting from the mesial papilla of 1.8, a crestal incision up to the distal papilla of 1.4 with the preservation of the papilla between teeth 1.5 and 1.4, and then a discharge towards the fornix in the mesial direction (Figure 2).

**Figure 3:**
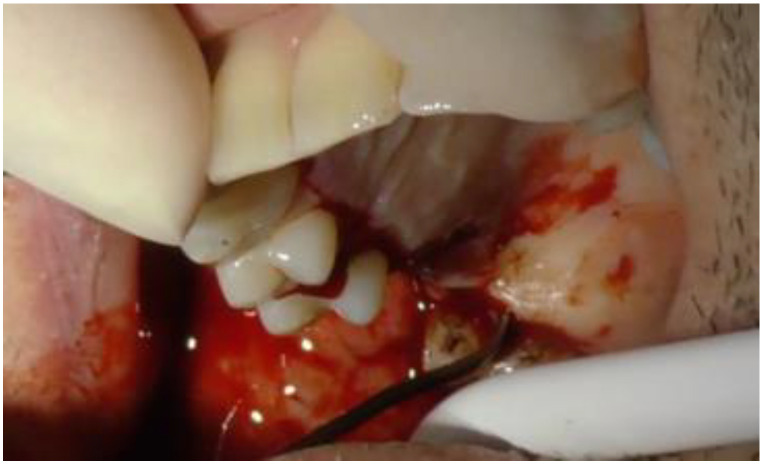
Detail of the design of the palatal flap.

On the palatal side, the incision extended from the base of the distal papilla of 1.8 to the base of the mesial papilla of 1.3 (Figure 3).

**Figure 4:**
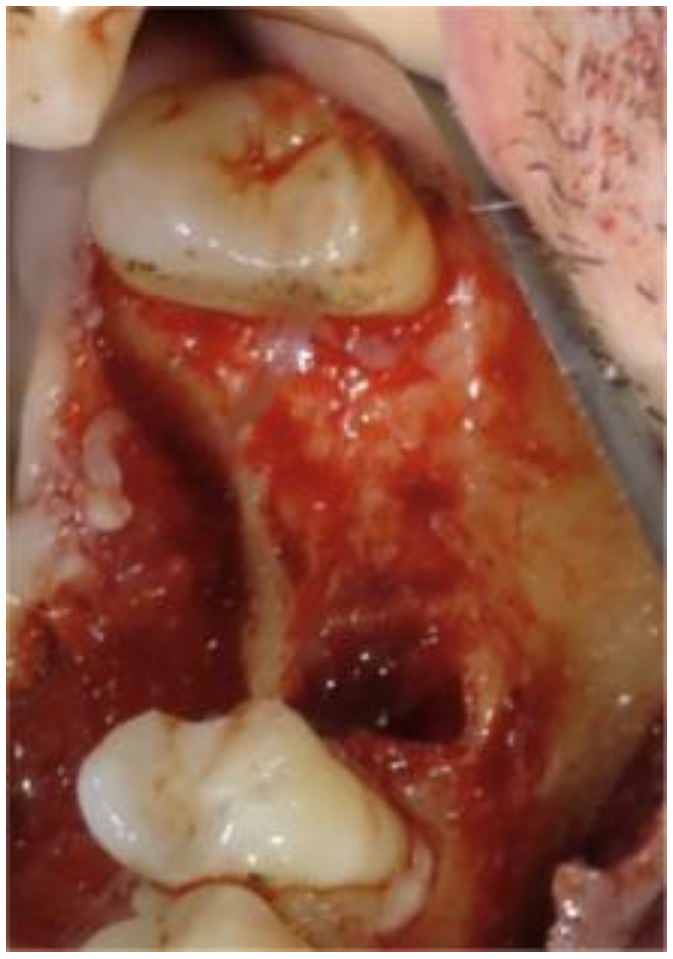
Crestal view of the bone after tooth extraction of 1.6. A small trabecula of alveolar bone was saved.

It was observed that the palatal alveolar bone of 1.6 was fully resorbed (Figure 4).

On the vestibular side, there was a bone fenestration at the mesial root apex caused by a granuloma.

**Figure 5:**
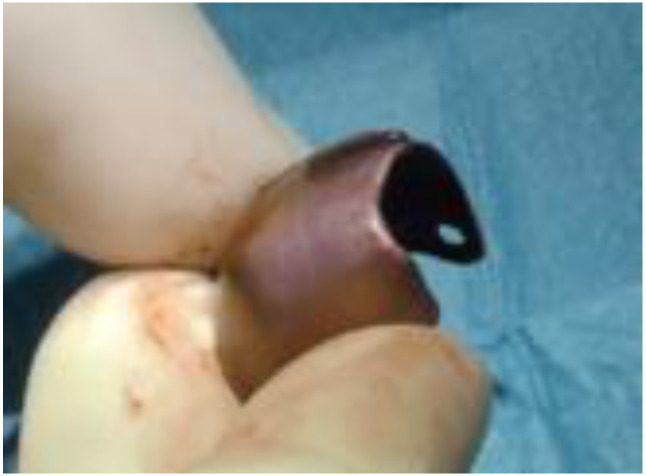
Titanium barrier.

After having curetted the site, the occlusive barrier (Figure 5) was tested to evaluate its ease of seating. The undercuts of neighboring teeth might require a particular insertion axis.

In addition, the flaps can make access to the tightening screws of the barrier challenging in terms of visibility. 

**Figure 6:**
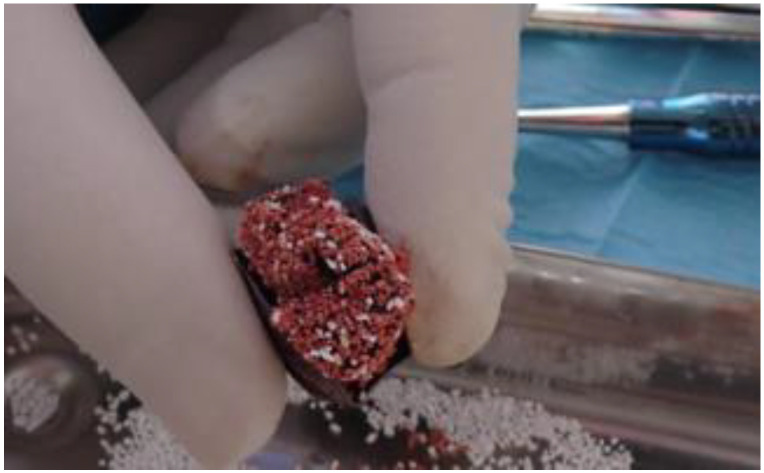
Barrier filled with clot and beta tricalcium phosphate.

The barrier was filled with the clot enriched with beta-phosphate and inserted into the site to be regenerated (Figure 6).

The barrier was fixed with appropriate screws.

**Figure 7:**
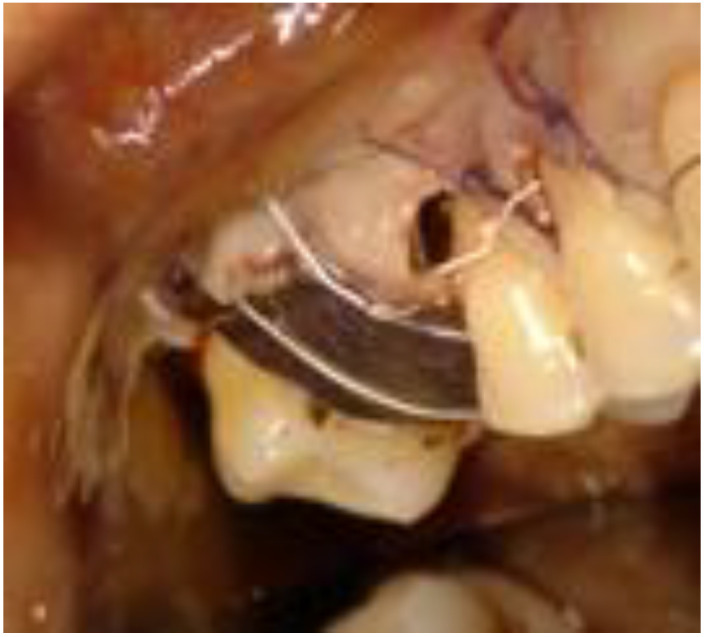
Detail of the sutures: resorbable for the release incisions and in PTFE to keep the flap close to the barrier.

The flap was sutured at the release incisions with 4-0 absorbable sutures to achieve primary intention healing. In correspondence with the barrier, the flap was approached and maintained with horizontal mattress sutures with 3-0 PTFE threads (Figure 7).

The patient was advised to respect home hygiene rules and was discharged.

After 14 days, the stitches were removed and the barrier was sanitized with a solution of hydrogen peroxide and distilled water in the proportions of 1:2 and with chlorhexidine at 0.20%. The applications of disinfectants took place via a syringe thanks to a vestibular housing created to sanitize the inside of the barrier during the healing period. The sanitizing washes were repeated every 10 days until the barrier was removed.

After 6 months, the barrier was removed.

Under local anesthesia, de-epithelialization of the peri-barrier mucosal invagination was carried out. Small incisions were made to access the fixing screws where necessary.

Then, after removing the screws, the barrier was removed.

**Figure 8:**
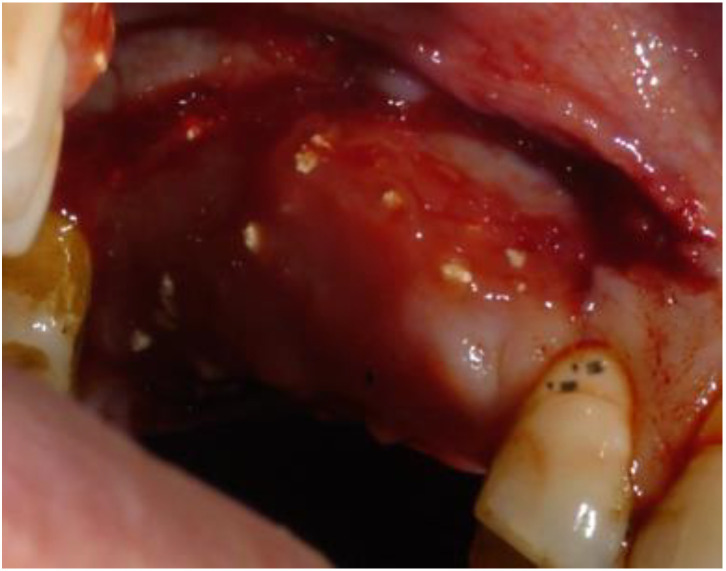
View of the newly formed tissue at the time of removal of the barrier. The granules of beta tricalcium phosphate can still be observed.

**Figure 9:**
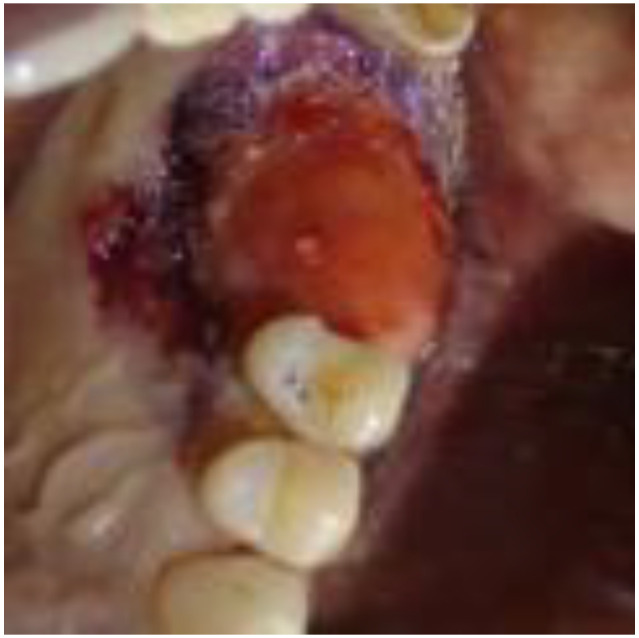
Detail of the application of the tissue glue to keep the tissues joined in situ.

An abundant, highly vascularized, hard-elastic tissue formed within the barrier (Figure 8). The newly formed tissue retained some beta-tricalcium phosphate granules. In correspondence with the distal papilla of 1.5, there was appreciable keratinization of the tissue, which indicated an initial tissue maturation. The flaps were left in place and maintained with tissue acrylic glue (Periacryl^®^ by GluStitch Inc., Delta, BC, Canada) (Figure 9).

A control CBCT was performed after two months. Since the interpretation of the radiological data showed a sufficient increase in bone volume, but it was not adequately mature, it was decided to wait for 8 months before carrying out the implant surgery.

**Figure 10:**
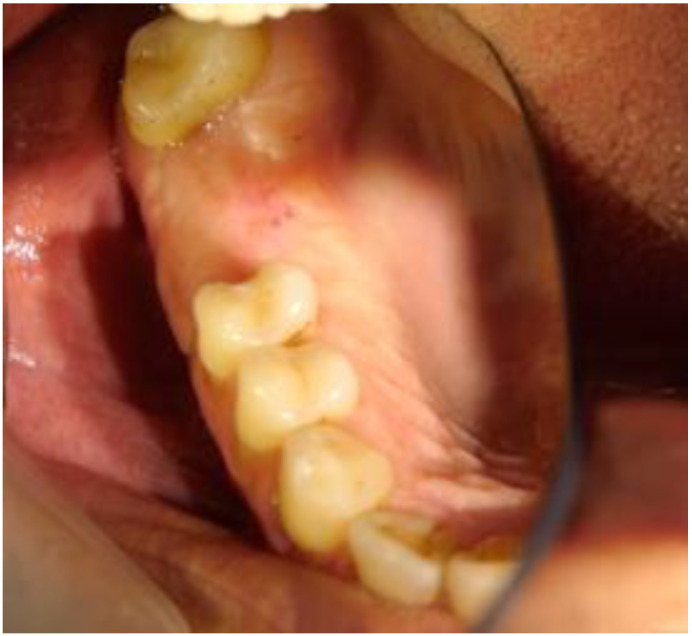
Appearance of the tissues after eight months of healing. A very well represented crest can be observed.

After 8 months, clinical control showed that the tissues had reached a high degree of maturation (Figure 10).

Another radiological check was carried out with a CBCT after 16 months, and it was decided to proceed with the insertion of the implants. 

In August 2020 (18 months after the GBR), the implants were placed.

The decision to apply the implants after 18 months was related to the difficulty of scheduling appointments in the COVID-19 era. Lockdown and, subsequently, the COVID-19 infection contracted by the patient, postponed the session for the insertion of the implants.

**Figure 11:**
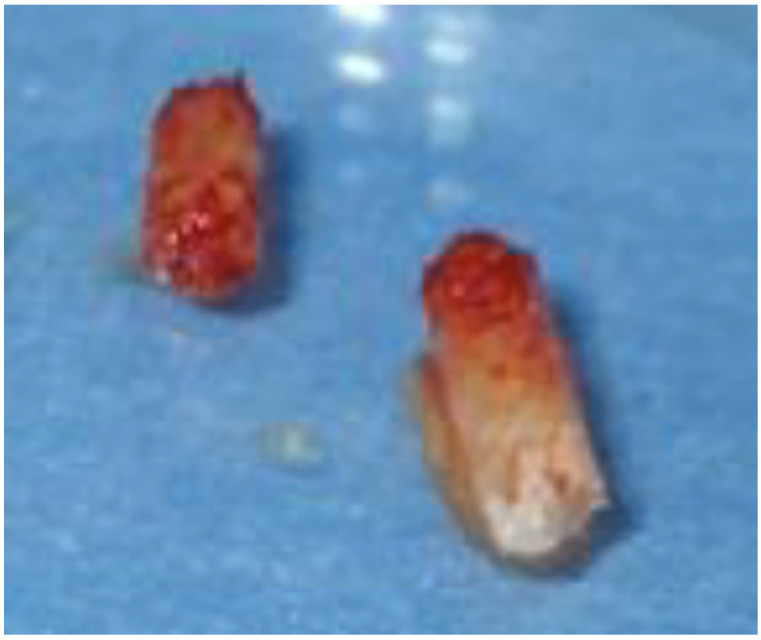
Bone carrots.

Under local anesthesia with articaine 1:100, a full-thickness envelope flap with a crestal incision with no proximal releases was incised. Two bone samples were obtained for a histological examination (Figure 11).

Two implants (EOS-Implant^®^), 4.5 mm in diameter and 10 mm in height, were inserted to sites 1.6 and 1.7. Both implants were inserted with a torque of 35 N.

Once the implants had been inserted, the Implant Stability Quotient (ISQ) was evaluated by analyzing the Resonance Frequency with a ISQ-Osstell ISQ Module^®^ (W&H Dentalwerk Bürmoos GmbH—Bürmoos Austria). Two measurements were performed: one mesio-distal and one bucco-palatal.

After 6 months, the implants were exposed, the ISQ was measured (average value 68.5 for the implant positioned in 1.6, and average value 65.5 for the implant positioned in 1.7), and it was decided to proceed with the temporary prosthesis in the infra-occlusion of the implants to functionalize the peri-implant bone.

It was decided to proceed with the measurement of the ISQ for 10 months.

**Figure 12:**
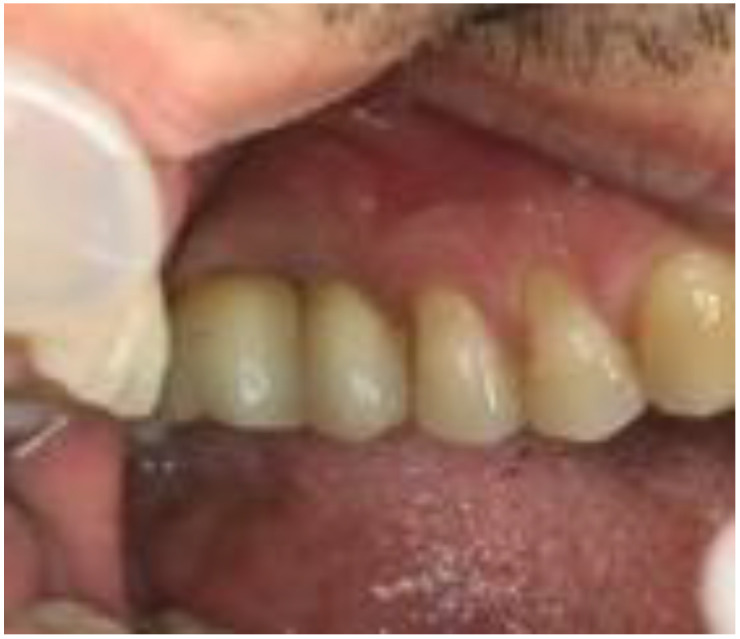
Final rehabilitation. It was preferred to finalize the case with a premolar 1.6.

Once the suitable ISQ had gained the optimal value (average value 72.5 for the implant positioned in 1.6, and average value 72 for the implant positioned in 1.7), the final prosthesis of the two implants was carried out (Figure 12).

### 2.2. Radiographic Observations

The CBCTs were all performed with a Planmeca Promax 3D^®^ machine. A 3D reconstruction was made (Implant 3D^®^ by Medialab S.p.A., Cambridge, MA, USA).

Three CBCTs were performed at different times: the first one at T_0_ (baseline) allowed for the planning of the entire implant-prosthetic rehabilitation procedure; the second (8 months after the GBR) allowed for verification of the regenerated bone maturation; the third CBCT (16 months after GBR) highlighted the maturation of the bone tissue and made it possible to plan the implantology.

**Figure 13:**
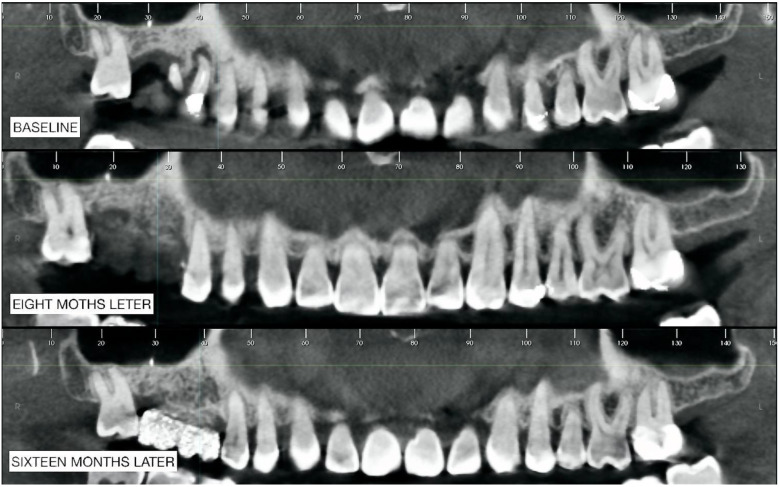
Comparison of panoramic cuts.

Comparing the panoramic images (Figure 13), a vertical bone augmentation was undoubtedly obtained. In zone 1.7, the radiopacity increased, indicating bone maturation (corticalization).

Furthermore, in zone 1.6, there was certainly an overall increase in radiopacity with an increase in corticalization. What was observed was an increase in radiolucency in the third CBCT in the apical area of 1.6.

**Figure 14:**
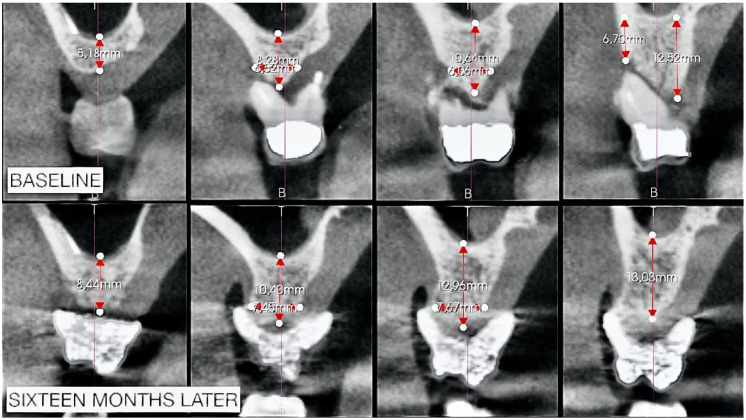
Measurements applied to the sections corresponding to teeth 1.7 and 1.6.

The sections of Figure 14 were obtained starting from the apical radiopaque landmark of 1.7 up to 15 sections in the mesial direction. From these images, it can be assumed that there was an increase in both vertical and horizontal volume with a bone that appeared more radiolucent at the first check (8 months later) instead of more radiopaque at the second check (16 months later); the latter being a sign of more significant bone maturation.

We compared (Figure 14) the CBCTs to the baseline and 16 months later in sections 1, 6, 10, and 12. These correspond to the edentulous area of 1.7 of the landmark, and the other sections correspond to the area of greatest alteration for the extraction of 1.6.

An evident gain of bone can be observed from the measurements performed, which, in the vertical direction, also reach about 3 mm.

### 2.3. Histological Analysis

The histological exam of the regenerated sites (1.6 and 1.7) evidenced areas of medullary bone with focal regenerative aspects of appositional type with significant gaps in the bone marrow adipose tissue.

**Figure 15:**
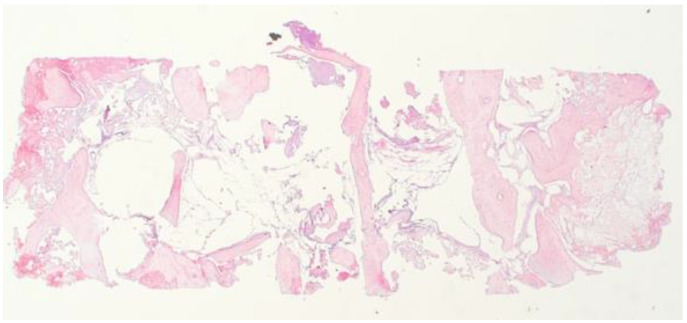
3× magnification.

**Figure 16:**
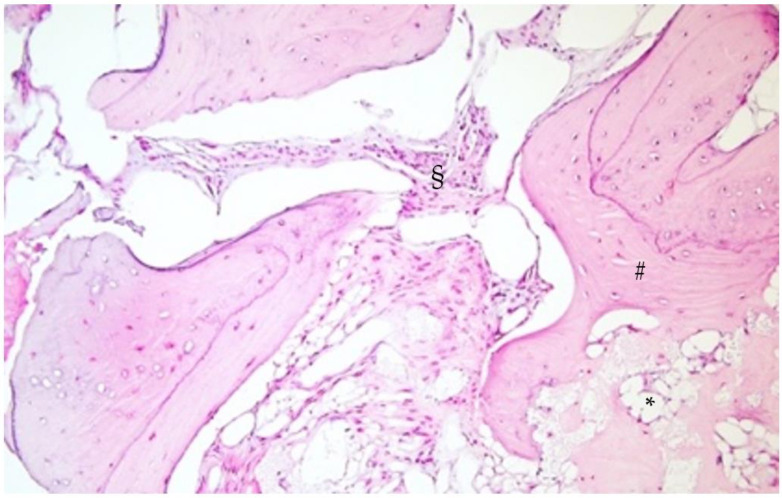
30× magnification. * Adipose tissue, § medullary bone, # mature bone.

The regenerated bone showed areas of maturity associated with remodeling aspects, highlighting a bone metabolism similar to the native bone. No granules of heterologous material were evidenced (Figure 15 and Figure 16), nor were they evidenced after a more comprehensive histological exam.

The high density of osteocytes confirmed the presence of a recently matured bone with a lamellar appearance.

### 2.4. ISQ Measurements

**Figure 17:**
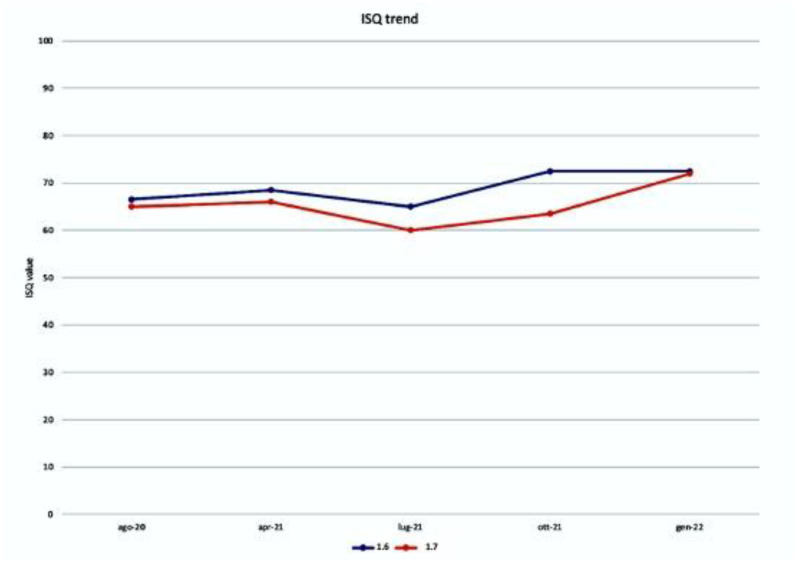
ISQ trend from the moment of implant application up to the moment of prosthetic finalization.

The measurements of the Implant Stability Quotient, the average of which is shown in the table (Figure 17), for both sites are the following: August 2020, at the time of insertion of the implants, the value was 68 b and 65 m for the implant in zone 1.6, of 65 b and 65 m for the plant in zone 1.7; April 2021, at the time of reopening the value was 70 b and 67 m for the plant in zone 1.6, of 67 b and 65 m for the implant in zone 1.7; July 2021, at the time of application of the provisional crowns in infraocclusion, the value was 67 b and 73 m for the implant in zone 1.6, of 67 b and 53 m for the implant in zone 1.7; October 2021, at the time of the inspection the value was 70 b and 75 m for the implant in zone 1.6, 67 b and 70 m for the implant in zone 1.7; January 2022, at the time of the final check the value was 70 b and 75 m for the implant in zone 1.6, 72 b and 72 m for the implant in zone 1.7.

## 3. Discussion

The clot plays a key role in GBR, and its protection during its maturation process is essential to obtain good bone quality for successive implant insertion [16].

In a comparative study of GBR between bone grafts vs. Ti barriers with only a clot, Molly et al. indicated that, although less final bone volume was achieved with the clot-only method, the resorption of peri-implant marginal bone was more stable over time in newly formed bone than in grafted bone [17].

Even if autologous bone transplantation is currently considered the gold standard in clinical practice, this approach presents variable reabsorption timing [18,19,20,21,22], and the procedure requires surgical techniques at high risk of complications [23].

Among the most used bone substitutes, xenografts, particularly the inorganic bovine bone matrix (ABBM), do not guarantee complete resorption in favor of a newly formed bone tissue for a very long time [24,25,26].

The phases of osseointegration and maturation of the bone around the implant have been extensively documented [27].

In addition, several studies have focused particular attention on the implants’ osteocyte index. The presence of osteocytes is an index of bone reactivity [28,29,30].

According to our histological samples, there were gaps in the adipose marrow, and osteocytes were very well represented in the areas of bone obtained by apposition.

According to Franchi et al., the masticatory load exerted on the implants after osseointegration can affect the biological turnover of the peri-implant bone up to 1 mm from the surface of the implant itself, with both resorption and osteogenesis processes [31].

This indicates the importance of implant placement when surrounded by bone responsive to mechanical stimuli.

Resonance frequency (RFA) analysis has long been used to measure the implant stability quotient (ISQ) and how this varies as a function of time and chewing load [32,33,34].

In this case, to evaluate the quality and behavior of the bone obtained after placing the implants, we measured the ISQ.

To our knowledge, few published studies have compared the implant stability between implants placed in native and regenerated bone.

Janyaphadungpong et al. evaluated the ISQ trend on twenty-two implants, fifteen placed in the mature bone and seven in mature bone, but with the simultaneous regeneration of dehiscence, limited to the period of osseointegration. They observed a more significant reduction in the ISQ at 2 and 4 weeks in the regenerated bone group [35].

A very interesting study was conducted by Farias et al. after obtaining a horizontal volume increase with the tenting screw technique in the posterior mandible using allogeneic material with blood products. The ISQ was measured to evaluate the primary and secondary stability of the implants [36].

Vallecillo-Rivas et al. compared the primary and secondary stability of the implants placed in the regenerated bone to the native one after tooth extractions: 30 implants inserted in the non-regenerated bone 6 months after extractions, and 30 inserted 6 months after the xenograft graft after exodontia. ISQ was measured at implant placement, after 8 weeks and 12 weeks. A statistically significant difference was found between the implants: excellent stability was achieved in the implants placed in the native bone; however, those placed in the regenerated bone showed adequate primary and secondary stability for the prosthetic loading [37].

The studies cited, although interesting, are limited to evaluating the difference in ISQ stability between primary and secondary implantation.

Deli et al. compared the trend of the implant ISQ at the time of implant insertion (primary stability) and after 4 and 8 months from the occlusal load of three implant groups, according to the regenerated and non-regenerated bone. The implants were placed in post-extraction sockets that spontaneously healed after 6 months, in post-extraction sockets that healed with the socket-preservation technique after 6 months, and in post-extraction sockets that healed with the socket-preservation technique after 12 months. The authors found that a level of stability compatible with implant success had been achieved in all conditions. However, the best performance of ISQ was that achieved by implants inserted in the regenerated bone at 1 year. The interesting fact to report is that the authors carried out socket-preservation without applying any graft to the socket, but only used the d-PTFE barrier to isolate the post-extraction socket from the surrounding soft tissues [38].

Chipaila et al. [39] measured ISQ 6 months after performing a sinus lift with simultaneous implant placement using an equine collagen sponge and clot as filling material. They noted that the ISQ stabilized between the fourth and sixth months. Therefore, biologically active bone was observed around the implants [40].

Previous studies on the use of perforated titanium membranes have shown good clinical successes, but the presence of holes exposed the membranes to a greater risk of infection [13,41].

In this case, we can make two observations from the ISQ data collected. The first observation that emerges is that of the substantial stability between the application of the implants and the moment of reopening, but with unsafe values to proceed with a definitive prosthesis and then proceed with a temporary prosthesis to be able to monitor the stability over time. The second interesting observation is that after a decrease in value, an increase is obtained up to obtaining safety values for the application of definitive prostheses.

Clinical and instrumental data that emerged from this case report (with its intrinsic limitations) suggest that a bone surrounds the implants inserted, obtained thanks to the regeneration of the enriched clot, being highly specialized and responsive to stimuli.

Further studies are needed to prepare a comparative analysis with a control group: although case reports have a low level of evidence, they are essential to set and plan more complex trials.

## 4. Conclusions

The presented case documented a GBR with good quality of bone obtained, the absence of heterologous material, and the masticatory function to rehabilitate with the absence of bone, implant, and prosthetic complications at the follow-up. 

Further studies and cases with longer follow-up times will be needed to confirm these data.

The clot plays a key role in bone regeneration, and tissue engineering will orient studies in the search for a material that implements its role in fulfilling highly specialized and responsive bone.

## Data Availability

Data is unavailable due to privacy. Please contact the corresponding author for information related to the article.
